# Toward understanding social cues and signals in human–robot interaction: effects of robot gaze and proxemic behavior

**DOI:** 10.3389/fpsyg.2013.00859

**Published:** 2013-11-27

**Authors:** Stephen M. Fiore, Travis J. Wiltshire, Emilio J. C. Lobato, Florian G. Jentsch, Wesley H. Huang, Benjamin Axelrod

**Affiliations:** ^1^Department of Philosophy, Cognitive Sciences Laboratory, Institute for Simulation and Training, University of Central FloridaOrlando, FL, USA; ^2^Institute for Simulation and Training, University of Central FloridaOrlando, FL, USA; ^3^Department of Psychology, University of Central FloridaOrlando, FL, USA; ^4^iRobot Corporation, BedfordMA, USA

**Keywords:** human–robot interaction, intention recognition, social signals, social cues, social presence, theory of mind, emotion attribution

## Abstract

As robots are increasingly deployed in settings requiring social interaction, research is needed to examine the social signals perceived by humans when robots display certain social cues. In this paper, we report a study designed to examine how humans interpret social cues exhibited by robots. We first provide a brief overview of perspectives from social cognition in humans and how these processes are applicable to human–robot interaction (HRI). We then discuss the need to examine the relationship between social cues and signals as a function of the degree to which a robot is perceived as a socially present agent. We describe an experiment in which social cues were manipulated on an iRobot Ava^TM^ mobile robotics platform in a hallway navigation scenario. Cues associated with the robot’s proxemic behavior were found to significantly affect participant perceptions of the robot’s social presence and emotional state while cues associated with the robot’s gaze behavior were not found to be significant. Further, regardless of the proxemic behavior, participants attributed more social presence and emotional states to the robot over repeated interactions than when they first interacted with it. Generally, these results indicate the importance for HRI research to consider how social cues expressed by a robot can differentially affect perceptions of the robot’s mental states and intentions. The discussion focuses on implications for the design of robotic systems and future directions for research on the relationship between social cues and signals.

## INTRODUCTION

The infusion of robots into society has expanded from industrialized environments to increasingly complex operations and even personal service contexts (Jones and Schmidlin, 2011). Robots are now engaged in human–robot interaction (HRI), not just in industrialized settings, but also in healthcare (e.g., Mutlu and Forlizzi, 2008), education (e.g., Saerbeck et al., 2010), therapeutic contexts (e.g., Ferrari et al., 2009; Robins et al., 2009), and in military (e.g., Yamauchi, 2004) and civil service operations (e.g., Murphy, 2004). This has created a tremendous amount of research addressing technological issues, ranging from robotic movement and control in engineering, to modeling cognition and social behavior in computer and cognitive science. One of the more pressing challenges regarding HRI is social cognition and the development of social capabilities in robots. Many robots still lack the ability to exhibit non-verbal social cues in ways that humans find natural and easy to understand, which is problematic for action and intention understanding (e.g., Breazeal, 2004). As the level of collaboration between humans and robots increases (e.g., in human–robot teamwork), these facets of interaction are increasingly critical because they directly impact a robot’s ability to effectively coordinate and cooperate with humans (Dautenhahn, 2007a). As such, research is needed to develop social-cognitive models supporting the ability of robots to interpret social cues so as to understand the intentions behind such actions, as well as understand how to display the appropriate social cues that can signal its own intentions to a human (see Breazeal et al., 2005).

The past decades have seen a number of interdisciplinary approaches addressing this problem, including social robotics (e.g., Fong et al., 2003) and social signal processing (SSP; Vinciarelli et al., 2009, 2012). At a general level, such research examines how robotic systems can interact with humans in a natural way. This requires a sophisticated form of social cognition where robots and people are able to infer each other’s intentions and attribute meaning to actions. In this context, our stepping-off point for the experiment reported in this paper is research in *social signals*. Generally, the study of social signals is a multidisciplinary field developing conceptual analyses, tools, and technologies that allow computer systems, such as robots, to be able to correctly perceive, accurately interpret, and appropriately display what we call *social cues* and translate these into *social signals* (Vinciarelli et al., 2009, 2012; Pantic et al., 2011). We focus on a small aspect of this research to examine a subset of cues exhibited by a robotic system and how this social display is interpreted by humans; that is, how these cues are transformed into signals by humans interacting with the robot. Importantly, we also consider how people both perceive the robot as an entity in a shared space and perceive the robot as a social agent capable of perceiving the person.

The purpose of our research is to examine the social cues associated with mental state attribution, or how we understand the mind of others during interaction. Specifically, our goal is to understand the relationship between the manifestation of *social cues* and how these are translated to *social signals* that lead to an ability to infer the intentions of robotic agents. The outcomes of such research are two complementary aspects of a social signaling framework. First, we hope to garner a better understanding of the cues associated with particular mental state attributions. At a theoretical level, this will help provide foundational knowledge on social signaling and specific foci for attention to salient cues and their meaning. Second, at a practical level, this understanding can be used to make recommendations for the requirements of robots exhibiting particular social cues. For example, speed of approach (a social cue), can be used to convey a sense of urgency (a social signal). From this, we will be able to recommend the parameters driving these attributions, and the metrics within these parameters for robotic design in HRI.

We view *social cues* and *social signals* as the basic conceptual building blocks of the kind of social intelligence necessary for interaction between humans and robots. We base this on the multidisciplinary approach of SSP (Vinciarelli et al., 2009, 2012) in which the goal is to understand social cognition as it relates to how social cues and signals are interpreted. Social intelligence is viewed as the skilled use of interpretation and expression in support of interactions. *Social cues* can be defined as biologically and physically determined features salient to observers because of their potential as channels of useful information. These can be operationalized as short, discrete sets of physical and behavioral features in a person or group (Streater et al., 2012). *Physical cues* consist of aspects of physical appearance and environmental factors, such as the distance between a social agent and an observer. By contrast, *behavioral cues* consist of non-verbal movements, actions, and gestures as well as verbal vocalizations and expressions using the body and face. Examples include eye movements, head nods, smiles, laughter, and arm positioning (Pantic et al., 2011; Vinciarelli et al., 2012). We consider *social signals* as semantically higher than social cues and as being emotionally, cognitively, socially, and culturally based. More specifically, social signals can be operationalized as meaningful interpretations based on mental states and attitudes attributed to another agent (Streater et al., 2012). Examples include dominating, flirting, attention, empathy, politeness, or agreement (Pantic et al., 2011; Vinciarelli et al., 2012). We maintain that this distinction between *social cues* and *social signals* is warranted given that contextual and cultural factors will influence how social cues are interpreted. As such, separating the social cue (i.e., the action itself) from the social signal (i.e., how an action is interpreted), provides an important level of clarity for research in HRI. From the standpoint of theory development, this allows us to more carefully distinguish between, and elaborate upon, how physical activities are differentially translated into meaning, dependent upon the context or the culture of the interaction. Further, from the standpoint of technology, this distinction provides more precise guidance for modeling social cognition in varied contexts and for specifying the types of cues robots must exhibit that are most likely to produce particular types of signals as interpreted by humans (Streater et al., 2012).

In this context, the aim of this paper is to examine how social cues expressed by a robot affect the human understanding of robot mental states (i.e., signals), so as to interpret and understand robot intentions. To meet this objective, we ground this work within the framework of human social cognition, with a particular focus on research regarding human attribution of social qualities to machines. The present research examined the effects of the *social cues* (gaze and proxemic behavior), as instantiated in a non-humanoid robot platform, on *social signals*, operationalized as perceived social presence and emotional state attributions to the robot. Accordingly, research regarding the importance of social cues for understanding intentions in both human–human interactions (HHIs) and HRIs is next briefly reviewed. In this, we weave together a set of theoretical concepts we argue can contribute to understanding intentions during HRI.

Currently, perspectives on social cognition in humans are aligned in their view that an integrated set of mechanisms is employed in understanding both the self and the intentions and mental states of other social agents (e.g., Baldwin and Baird, 2001; Beer and Ochsner, 2006; Chiavarino et al., 2012). There is also increasing support from a number of disciplines that this is done using automatic and/or deliberate processes (see Lieberman, 2007; Apperly and Butterfill, 2009; Bohl and van den Bos, 2012). Typically, the purpose of employing such social cognitive mechanisms is that in gaining an understanding of others mental states, one will be able to respond and interact appropriately as well as anticipate future states (e.g., Przyrembel et al., 2012).

When applied to human–machine interactions, human social cognitive mechanisms seem to elicit a tendency to treat machines as social agents. Interestingly, this tendency appears to be automatic and implicit (Nass et al., 1993; Reeves and Nass, 1996; Nass and Moon, 2000; Takayama and Pantofaru, 2009; Mumm and Mutlu, 2011). However, several recent HRI studies have shown that, within some contexts, participants will explicitly consider *and* treat a robot as an autonomous social agent (Saulnier et al., 2011; Kahn et al., 2012a,b). Therefore, using a framework of how humans process human social signals and apply it to HRI seems promising.

We argue that to apply social cognition to HRI, SSP should be leveraged to better understand the role of social cues and signals in influencing one’s perceptions of a robot as a social agent. As described earlier, SSP is a rapidly developing field that aims to understand social cognition and the ways in which social cues and signals can be interpreted and conveyed by computerized systems (Vinciarelli et al., 2009, 2012). Recent efforts have built on this as a foundation for explicating social dynamics in the context of HRI for both robot and human social perception and interaction (Bockelman Morrow and Fiore, 2012; Streater et al., 2012; Lobato et al., 2013; Wiltshire et al., 2013a). Understanding the exchange of social cues and the resultant social signals just in HHIs is a challenge because they come in an almost innumerable quantity of combinations that are context dependent (Vinciarelli et al., 2012). Nonetheless, as robotic systems increasingly enter society, research is needed to better understand this exchange as it occurs between humans and robots. While there exists some research on the types of physical and behavioral cues conveyed by robots, even if such cues were intended or programmed to communicate a certain signal, less work has explored how those signals are actually perceived by humans (see Cabibihan et al., 2012; DeSteno et al., 2012, for examples). Such efforts are necessary given that an ultimate goal for the development of robotic teammates is for humans to intuitively understand the intentions of the robot during a given interaction in order to facilitate effective communication and shared situation understanding (e.g., Klein et al., 2004; Pezzulo, 2012).

For the successful exchange of social signals between humans and robots, we suggest that humans must consider the robot as a social agent so as to perceive the cues as social in nature and then work to recognize intentions. In the context of HRI, this is thought of as the degree to which the robot is perceived as socially present**(Jung and Lee, 2004). *Social presence* can be defined as the degree to which an agent perceives being in the company of another social agent (Harms and Biocca, 2004) and is thought to be key for gaining a sense of accessibility or understanding of another agent’s intentional, psychological, and emotional states (Biocca and Harms, 2002). Further, social presence is also the degree to which a technology is able to scaffold and elicit a sense of social interaction (de Greef and Ijsselsteijn, 2000; Fiore et al., 2003) and more pertinently, is a theory of how technology may affect, distort, and enhance certain social–cognitive processes in humans (Biocca and Harms, 2002).

In short, social presence theory aims to describe the processes through which humans are able to understand the intentions of others, with an emphasis on how this is done during social interactions with artificial agents and/or technologically mediated human interaction (Biocca and Harms, 2002). As such, examining the perceived social presence of a robot enables assessment of the degree to which humans understand its intentions. For research in HRI, we suggest that an important question to consider, in the context of social–cognitive mechanisms, is the degree to which robots are able to convey a sense of social presence as a function of the social cues they display and the social signals that the cues convey to an observer.

Given the upsurge of distributed and virtual interaction in the early part of the twenty-first century, a tremendous amount of research has examined the many ways social presence has impacted human behavior. This ranged from studies of how distribution alters teamwork (e.g., Driskell et al., 2003) to how interaction with avatars changes behavior (e.g., Slater et al., 2000). As part of this research, Harms and Biocca (2004) developed the *networked minds social presence inventory *(NMSPI) to examine perceptions of self and another when interacting through technology. The NMSPI asks participants a series of questions about their own perceptions of another entity as well as to assess how the other entity perceives the participant, while taking into account the symmetry of the interaction (Abeele et al., 2007). For example, two questions from this scale are “It was easy for me to understand (my partner)” and “(My partner) found it easy to understand me.” The remaining questions in this scale follow the same structure, in that pairs of questions have the opposite subject and object. In instances where the participant is the object, the questionnaire asks participants to judge the mental states of the other entity during the interaction. In this way, the NMSPI prompts participants to make mental state attributions, that is, to engage in theory of mind mentalizing, about the capabilities of the other entity. Thus, the NMSPI allows for the deliberate employment of social cognitive mechanism to allow for the investigation of attributions of the mental states of another agent.

In short, an important distinction within the NMSPI has to do with attributions about the self as opposed to attributions about some other. Although this distinction was not made explicit in the original development of the NMSPI measure, this enables researchers to analyze responses along the symmetry of self and other attributions within an interaction (Abeele et al., 2007) in order to uniquely examine the different ways social presence is related to cues and signals. Importantly, components of this scale have been used previously in HRI research (Leite et al., 2009). In this work, Leite and colleagues were interested in changes in perceived social presence over long-term HRIs. The researchers examined children’s perceived social presence of an iCat robot in a game playing context over a 5-week period of time. Their results showed a decrease in perceived social presence over time, though the authors suggest that the limited context of the interactions and the relatively limited capabilities of the robot were not sufficient to maintain the appearance of a socially present agent. Nonetheless, this is relevant because it shows that the NMSPI, though developed originally to examine social presence of another human, can also be a useful instrument in exploring the perceived social presence of a distinctly non-human entity like a robot.

While the NMSPI is a measure of social presence, we suggest that, in the context of HRI, the distinction between self- and other-attributions, inherent to the measure, is a potentially useful research approach. From this, we can examine the relationship between social cues and signals when a human is interacting with a non-human entity by using the other-attributions as a means of assessing how social cues are translated into social signals. In juxtaposition, we are able to apply the NMSPI as a means of measuring the degree of intention understanding attributed to participants’ selves regarding the robot.

Generally, within HHI, humans make attributions about another as to their cognitive and/or their emotional states. Methods such as those described above focus more on the cognitive facets of intention understanding. But an important component of mental state attribution and understanding the intention of another, is how that entity is feeling (i.e., the entity’s affective/emotional state). Making mental state attributions about another’s emotional states is an essential part of the process for understanding the intentions behind a given action and is often done from a limited set of observable social cues (e.g., Olsson and Ochsner, 2008; Mier et al., 2010). Research in this area examines how factors such as context, expressions, gestures, facial features, etc., drive the attribution of certain social signals, including emotional and mental states, and the results these have for a given interaction (Forbes and Grafman, 2013).

While emotion attribution is clearly related to understanding the intentions of humans (see Mier et al., 2010), we suggest it may also be important for HRI. Relatedly, Bethel and Murphy (2008) highlighted three primary issues relevant to advancing emotional expression by “appearance-constrained” robots using non-facial and non-verbal cues. These include: “(1) the development of an accurate model of emotion generation; (2) how to measure emotional responses to the robot; and (3) how the robot can accurately convey its role and intent” (Bethel and Murphy, 2008, p. 91). The importance of understanding how humans attribute emotions to a robotic platform relates to all three of these issues raised by Bethel and Murphy. Taken together, these suggest that how humans respond emotionally to a robot will depend on their interpretation of the emotional state of the robot based on the cues it expresses. Thus, the perceived emotional states of a robot can be used by humans to augment their understanding of the robot’s intentions.

A large body of work has explored the underlying dimensions of emotional states. From a number of experiments, Russell (1980) derived the circumplex model of affect, which arranges emotional states at equidistant points around a two-dimensional space representing the orthogonal dimensions of valence and arousal. Parsing emotions apart from their semantic level of description (e.g., happy, sad, etc.) to these two underlying dimensions allows for consideration of the level of positivity/negativity (valence) and level of activity/inactivity (arousal) associated with a given emotion. Importantly, from this line of work came new measurement tools, relying on the semantic descriptions, which have often been used for assessing self-attributions of emotional states (Jacob et al., 1989), but also for measuring the emotions attributed to others’ facial expressions (Russell et al., 1989). Specifically, the circular mood scale (CMS; Jacob et al., 1989), originally developed and validated for self-assessments of one’s own mood, is an emotional state measurement tool derived from the circumplex model of affect that can also be used for the attribution of emotions to others. In addition to valence and arousal, a third dimension, representing intensity of mood, is intrinsic to the CMS. With regard to emotional state attributions, intensity can range from a neutral emotional attribution to a very strong attribution of a given emotion. In short, this is a single-item measure that allows participants to make an attribution using a graphical array of emotions.

Taken together, attributions assessed with the NMSPI can be seen as more cognitive in nature (e.g., that entity is attending to me) and those assessed with the CMS are more emotional in nature (e.g., that entity is angry). Because emotion is a foundational element of social interaction, and, often, a driver of mental state attribution, we suggest that in HRI, it is essential to examine both cognitive and emotional mental state attributions made to a robot as a function of cues displayed by the robot. In doing so, we are able to assess the resulting social signals (both cognitive and emotional).

Recent research in robotics has addressed some of the complexities inherent in intention understanding by examining if humans can accurately interpret “minimal cues.” These are described as “behaviours that rely on only a few simple physical capabilities present in (or that can be easily added to) most robots” (Saulnier et al., 2011, p. 79). Such research has focused on relatively simple social interactions. For example, in their study of interruption behaviors, Saulnier et al. (2011) studied what was referred to as the “physical” aspect of social robotics. This was described as a fundamental layer of social interaction involving features such as robotic movement, interpersonal distance, and gaze. The researchers argued that these represent an important design feature for social robotics and that this “physical layer” needs to be considered in the design of robots in order to scaffold intention understanding. We see these as part of the broader theoretical framework of social cues and signals in that this physical layer represents the cues that humans translate into social signals that drive the ability to understand intentions through observed actions. As Saulnier et al. (2011, p. 79) noted, action understanding forms an important aspect of interaction in that even our own “actions are based on our expectations of how others will understand, interpret and ultimately respond to our interruption behaviours.”

In the present study, we applied the cues of gaze and proxemic behavior to an iRobot Ava^TM^ mobile robotics platform, a non-humanoid robot developed by the company iRobot, in order to determine how humans perceive these cues during a hallway navigation scenario. These behavioral cues were selected because of their established importance in both HHIs (e.g., Aiello and Aiello, 1974; Kleinke, 1986) and their increasing prevalence in HRIs (e.g., Pacchierotti et al., 2006; Takayama and Pantofaru, 2009; Walters et al., 2009; Mumm and Mutlu, 2011; Srinivasan et al., 2012).

Although our research was conducted in an experimental setting, the context for this research was an ecologically valid interaction. Participants were given the goal of traversing a hallway from one end to the other in which they encountered the robot. The use of a hallway crossing scenario has been used in HRI research previously, though mostly in service of creating effective robot navigation models (e.g., Pacchierotti et al., 2005), as opposed to collecting cognitive and behavioral responses by humans (e.g., Tsui et al., 2010). The robot in our study was programmed to traverse half of the hallway from the opposite direction of the research participant and branch off into an intersecting hallway. This scenario was chosen because it is one that would be very commonly encountered with mobile robots in many settings, including offices, hospitals, and other facilities (e.g., Mutlu and Forlizzi, 2008; Tsui et al., 2010). This ecologically valid interaction within an experimental setting allowed us to leverage the benefit of multiple trials along with a realistic experience (as opposed to a simulation setting). As such, we studied the impact of directly observable cues potentially driving mental state attribution. From this, we were able to have an indication of the relation between readily observable “physical cues” and the types of social signals arising when such cues are presented.

Gaze is one of the most frequently studied social cues in HHIs and has been shown to provide useful information that regulates a given social interaction and facilitates accomplishment of a task or goal (Kleinke, 1986). Generally, gaze can be defined as a visual behavior used as a cue for understanding the actions, intentions, and mental states of others (e.g., Kleinke, 1986; Baron-Cohen et al., 1995). For example, Castiello (2003) found that gaze plays a pivotal role in understanding the motor actions and intentions of another person. More recently, in social neuroscience, gaze in an interpersonal context has been demonstrated to be an expression of directly perceivable embodied intentionality that activates motor and perceptual resonance mechanism to facilitate the understanding of others actions and intentions (e.g., Gangopadhyay and Schilbach, 2012).

In HRI, social gaze behaviors of a robot have also been shown as an effective cue that humans can use to understand the behavior and intentions of a robot (e.g., Srinivasan et al., 2012). For example, when a robot provided minimal gaze cues, humans performed better on a task in which participants were to guess which object the robot was intending to pick (Mutlu et al., 2009).

At a fundamental level, a robot’s gaze type can be programmed according to different sets of rules, for example, oriented in the direction of the robot’s movement vs. oriented toward the nearest human, and this can have an effect on human behaviors. Mumm and Mutlu (2011), for example, demonstrated that gaze had differential effects on the physical distance humans kept between themselves and a robot dependent upon whether the humans had established positive rapport with the robot. Specifically, if a person had good rapport with a robot, then differing robot gaze behaviors did not affect the physical distance humans kept with the robot. However, if the person had not established good rapport with the robot (e.g., they did not like the robot), then robot gaze behaviors oriented toward the person increased the amount of distance the person maintained between himself and the robot.

Moving from human actions in response to a robot’s gaze to human attributions of the robot’s social presence and its emotional states, we apply the previous findings on the influence of human gaze and attributions of emotional states and present the following hypothesis:

*Hypothesis 1: the gaze type of the robot will differentially affect perceived social presence (H1_a_) and the emotional states attributed to the robot (H1_b_)*.

Proxemic behavior has traditionally been thought of as the ways in which humans utilize, structure, and position their body within the space around them in relation to other people and objects (e.g., Hall, 1963). Importantly, the proximity or spatial distance utilized during a given HHI can specify the type of interaction as intimate, personal, social, or public (Hall, 1963; Aiello and Aiello, 1974). Therefore, this cue has important implications for how a given person is both socially perceived and interacted with. Developmental research has shown that, as children grow older, they utilize greater proxemic distance during their interactions (Aiello and Aiello, 1974). More relevant for the understanding of intentions, proxemic behavior was found to be an important predictor of hostile and aggressive intentions on a simulated task (McCall et al., 2009).

In HRI, proxemic behavior has been extended to study varying social meanings as a function of the distance in which the robot comes within the human (e.g., intimate, personal, social, public; Pacchierotti et al., 2006). Recent efforts have gone so far as to develop computational taxonomies from which a bi-directional intention understanding can be derived as a function of the spatial distance between a human and a robot in a given interaction (Walters et al., 2009). Of course, human responses to robots as a function of the robot’s use of proxemics are context dependent, although research seems to broadly show that this can even impact humans’ affective states (e.g., Bethel and Murphy, 2008).

In our study, we manipulated proxemic behavior by varying the proximity the robot would reach toward the human during the hallway crossing scenario, either by speeding up and crossing the human’s paths during the encounter, or by providing the human more space and waiting until he/she passed. We predicted that the type of proxemic behavior would influence human attributions of the robot’s social presence and emotional states and thus we present the following hypothesis:

*Hypothesis 2: robot proxemic behavior will differentially affect perceived social presence (H2_a_) and the emotional states attributed to the robot (H2*_b_*).*

In their study investigating the effects of gaze and proxemics in HRI, Takayama and Pantofaru (2009) demonstrated that the comfortable minimal distance between a human and a robot depends, in part, on gaze behaviors by the robot, such that, when the robot’s gaze is oriented at a person’s face the comfortable distance between women and the robot increased, but, between men and the robot, the comfortable distance decreased. These studies show that these minimal cues interact, and warrant further study in social interactions between humans and robots. To expand on this literature, we postulated the following hypothesis for the interactive effects of robot gaze and proxemic behavior on human attributions regarding the robot’s social presence and emotional state:

*Hypothesis 3: robot gaze and proxemic behavior will have interactive effects on the perceived social presence (H3_a_) and the emotional states attributed to the robot *(H3_b_).

Most of the HRI research cited above has only examined either a single interaction between a robot and a human, or collapsed results from several interactions into a single measure. However, robots designed for use in social settings are intended to have multiple interactions with many of the same people over a period of time. Interacting with robots is a novel experience for most people, and attitudes are likely to change as novelty effects wear off. As such, one of the main themes in HRI research is to consider how people’s perceptions of robots change over time (Dautenhahn, 2007b).

Recent research that has examined the effects of repeated, or long-term, interactions with robots has shown that people who have previously interacted with a robot engage the robot differently than people who are interacting with the robot for the first time. For example, repeat visitors to a robot receptionist capable of digitally animated emotional expressions were more likely to have longer interactions with the robot than those who were interacting with it for the first time (Gockley et al., 2005) and less likely to interact with it during times it expressed a negative affect (Gockley et al., 2006).

More generally, research investigating changes in participant responses over multiple interactions is still relatively new (Leite et al., 2013). Given that interaction with robots is still often a novel experience for most people, we were interested in how perceptions of the robot would change over time. The current study adds to this literature by examining changes in participant perceptions over a series of repeated interactions in the hallway navigation scenario. While these interactions all occurred within a relatively short time-span, typically 60–90 min, measures were taken at multiple time points, allowing for the analysis of changes in mental state attributions and perceived social presence over time. Although this time period is probably not sufficient for novelty effects to be eliminated, we expected that time would have an effect on human attributions regarding the robot’s social presence and emotional state and thus pose the following hypothesis:

*Hypothesis 4: over the course of multiple encounters with the robot, perceived social presence will increase (H4_a_) and emotional states attributed to the robot by participants will change (H4_b_*).

In sum, the primary aim of the present research was to examine the effects of gaze and proxemic behavior on the perceived social signals attributed to the robot by human participants. We sought to extend the literature on gaze and proxemic behavior in HRI, in order to better understand how instantiation of social cues in a robot affects human perception of the robot as a social entity. Through examination of the perceived social presence of the robot and emotional state attributions as a function of the manipulated cues, our goal was to examine intention understanding in HRI. The secondary aim of this paper was to investigate how the behavioral and cognitive responses to a robot exhibiting these cues change over multiple interactions. In addition, we address our research question regarding the self- and other-attributions present within the NMSPI. We expect that, through examination of the differences between these two types of attributions, as a function of the social cues conveyed during the interaction, we will have a proxy for assessing the degree of perceived intention understanding attributed to the participants and to the robot. In short, this research will contribute uniquely to the HRI literature by simultaneously examining multiple social cues, investigating participants’ changes in perception across repeated interactions, and by using a single-item measure of affect that has not yet been utilized in HRI research.

## MATERIALS AND METHODS

### PARTICIPANTS

Seventy-four (74) volunteer participants (37 females and 37 males, *M*_age_ = 19.2 years) from a large southeastern university participated in this study. Treatment of human subjects was in accordance with the Declaration of Helsinki, was approved by the authors’ Institutional Review Board (IRB), and administratively reviewed and approved by the funding agency. Data from two participants were excluded due to technical problems relating to the robot not maneuvering according to the appropriate programming.

### MATERIALS

**FIGURE 1:**
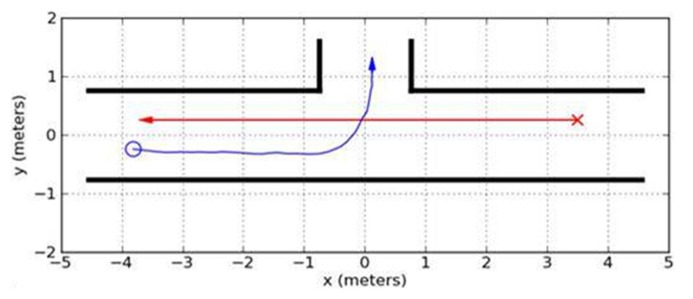
**Illustrative layout of the experimental space.** Participant starting point is denoted by the red “X” and the robot starting point is denoted by the blue “O.” Idealized paths to the trial end points are denoted for the participant and the robot by the red and blue lines, respectively.

**FIGURE 2:**
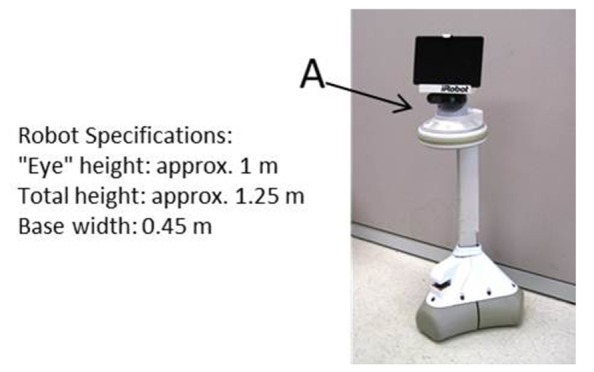
**iRobot Ava mobile robotics platform and physical specifications.** Primary robot sensors are denoted by “A.”

The experiment was conducted in a large laboratory space, using a 5 ft. by 30 ft. (approx. 1.5 m × 9.1 m) rectangular hallway constructed for this experiment (see **Figure 1**). An iRobot Ava mobile robotics platform designed and provided by iRobot, and programmed specifically for utilization in this experiment, was used (see **Figure 2**). A Hokuyo laser system was used for motion capture, and three Logitech webcams were used for video recording of the experiment. Two webcams were located above the experimental space, each one capturing half of the space from a “bird’s eye view,” and a third camera was placed at the mid-point of the hallway at approximately 1.5 m height, in order to capture participant behavior from a different perspective. Participants responded on a computer terminal outside the hallway to a number of subjective measures, described below.

The CMS (Jacob et al., 1989) was used to assess emotions participants would attribute to the robot. This measure presents participants with a circular figure surrounded by multiple words denoting eight affective states and eight simple drawings of faces depicting each emotional state. The graphic is derived from the circumplex model of affect, which arranges emotional states at equidistant points around a two-dimensional space representing the two orthogonal dimensions of valence and arousal (e.g., Russell, 1980). Valence is represented along the horizontal axis and arousal is represented along the vertical axis. A third dimension, representing intensity of emotional state, is also intrinsic to the CMS, reflecting the distance from the center that participants respond without regard to either valence or arousal.

Four subscales from the NMPSI, a validated measure of social presence (Harms and Biocca, 2004), were selected for this experiment. The subscales used were co-presence, attentional allocation, perceived message understanding, and perceived behavioral understanding. The survey was modified for the purpose of our study (e.g., references to “my partner” were replaced with “Ava” and the word “thoughts” in questions 13 and 14 were changed to “intentions”). Responses were given on a 5-point Likert scale (from “1” = “strongly disagree” to “5” = “strongly agree”). As detailed in the introduction, for the present purposes, the research question of interest regarding the NMSPI was the novel distinction of self- and other-attributions of social presence, rather than responses on the traditional subscales (for additional analyses of NMSPI, see Wiltshire et al., 2013b). Thus, we separated the questions into two subscales; those related to “self” and those related to “other,” and analyzed the data at the level of these two subscales.

### DESIGN

The basic design of the experiment was a 3 (gaze: congruent, human-oriented, variable; between subjects) × 2 (proxemic behavior: passive, assertive; within subjects) × 2 (measures taken after trial 1 and trial 6 for a given proxemic behavior; within subjects) mixed-model design. Each participant was randomly assigned to one of the gaze conditions and completed a total of 18 trials, six for the passive proxemic behavior condition and six for the assertive proxemic behavior (the final six trials examined computational factors related to robot navigation and are beyond the scope of this paper). The blocks of six passive and six assertive trials were counterbalanced in presentation, such that 50% of the participants completed the six passive proxemic behavior trials first and the other 50% completed the six assertive proxemic behavior trials first.

#### IVs

The independent variable of gaze had three levels, operationalized as the direction that the robot’s primary sensors were oriented. The robot’s sensors were installed on the robot in a way that – both by location (top) and function (rotating horizontally and independently from the robot’s base) – could be perceived by a human as the robot’s head or eyes (see **Figure 2**). The three levels of the gaze variable were congruent, human-oriented, and variable. *Congruent gaze* was defined as the head consistently being locked in the direction of the robot’s movement throughout the interaction. *Human-oriented gaze* was defined as the head consistently being oriented approximately at the head of the participant. *Variable gaze* was defined as an initial orientation of the robot’s head toward the participant’s head and then toward the navigation goal. Variable gaze was considered the most “natural” gaze behavior as it provided both the cue of sensing the human interactor and the cue of intended direction of travel.

Proxemic behavior had two levels and was operationalized as the degree of proximity the robot would allow the human in determining whether to cross paths or wait until the participant passed. The *passive* behavior slowed the robot and modified the path to the side of the hall to provide more space for the participant to pass in front of the robot. The *assertive* behavior sped up the robot and modified the path to “cut the corner” so as to pass in front of the participant.

#### DVs

Dependent variables examined in this paper include results of participant ratings on the NMSPI for each of the four times it was completed during the experiment: after trial 1, after trial 6, after trial 7, and after trial 12. Again, this was a first and second measurement for each of the proxemic behaviors parsed according to whether items represented self- or other-attributions of social presence. This novel approach for analyzing the NMSPI was used as a means of measuring the degree of intention understanding attributed to participants’ selves (subject) regarding the robot as the object (which we labeled “self”) as well as the degree they attributed to the robot (subject) with themselves as the object (which we labeled “other”). The additional dependent variables include the responses to the CMS along the dimensions of valence, arousal, and intensity across each of the four measurement times.

### PROCEDURE

After providing informed consent, participants were brought into the experimental space. First, they were seated at a computer terminal and provided a tutorial for how to respond to the CMS, as this was assumed to be a novel response method for participants. In this tutorial, participants were presented with the CMS and an adjective (e.g., JOYFUL) and asked to select a point on the graphic where they felt was most indicative of the word. They were informed that the space between two sets of terms represents the continuum of emotions between those sets, and that the space between the center and the perimeter represents an intensity dimension, such that the center point represents the most neutral or muted expressions of an emotion and the perimeter of the graphic represents the most intense expressions of an emotion. This training phase consisted of eight such stimuli.

After this phase, participants were brought to the “entry point” of the hallway. This is where they first encountered the robot. At this point, the video cameras were turned on. Participants were informed that they would be asked to walk toward the other end of the hallway when instructed to by the experimenter. The experimenter instructed the participants to walk toward the other end of the hallway when the robot began moving. After the first trial was completed, participants were asked to sit at the computer in order to fill out the measures. When responding to the CMS, participants were asked to select a point on the graphic to represent which mood or emotional state they felt the robot was exhibiting.

After completing the measures, participants were brought back to the entrance of the hallway and completed trials 2 through 6 before responding to the same measures for the second time. Trials 7 through 12 were conducted in the same manner as trials 1 through 6, with participants completing measures twice, specifically after trials 7 and 12. The participants then completed trials 13 through 18 (which studied other variables and are beyond the scope of this paper). Once the final trial was completed, participants were also asked to fill out a demographics form.

## RESULTS

### SOCIAL PRESENCE

We first report a series of analyses to understand the impact of proxemic behavior, gaze, and time on perceived social presence (hypotheses H1_a_, H2_a_, H3_a_, and H4_a_). All analyses were performed using SPSS version 21 with the alpha level at 0.05, unless otherwise stated.

The analyses examined social presence along two dimensions: self-attributions regarding the robot and other-attributions about the mental states of the robot. Items on the NMSPI with the participant as subject (e.g., “It was easy for me to understand Ava”) were categorized as “self,” and questions where the participant was the object (e.g., “Ava found it easy to understand me”) were categorized as “other.” Because of the modifications to some of the items on the NMSPI, the novel method for analyzing the findings (i.e., comparing self- and other-attributions), and the use of the scale across multiple measurement times, internal reliability alpha coefficients were calculated. These, and the mean values, standard deviations, and correlations are displayed in **Table 1**.

**Table 1:** Mean values, standard deviations, correlations, and internal scale reliability for NMSPI.

	*M*	SD	1	2	3	4	5	6	7	8
1. Passive time 1 self	3.70	0.50	(0.74)							
2. Passive time 1 other	3.48	0.74	0.48^***^	(0.90)						
3. Passive time 2 self	3.80	0.43	0.45^***^	0.07	(0.66)					
4. Passive time 2 other	3.80	0.63	0.27^*^	0.63^***^	0.34^**^	(0.87)				
5. Assertive time 1 self	3.77	0.40	0.16	-0.02	0.37^**^	0.08	(0.58)			
6. Assertive time 1 other	3.15	0.68	0.05	0.14	-0.03	-0.01	0.40^***^	(0.87)		
7. Assertive time 2 self	3.97	0.41	0.40^***^	0.06	0.50^***^	0.06	0.41^***^	0.10	(0.63)	
8. Assertive time 2 other	3.23	0.73	0.13	0.08	0.23	0.03	0.10	0.21	0.27^*^	(0.89)

**N *= 72. Internal scale reliability alpha coefficients are shown in parentheses on the diagonal. In addition, due to a lack of findings for the gaze manipulation, the values included here are not grouped along those dimensions.*

* *p *< 0.05; **p < 0.01; ***p < 0.001.

The reliability for self-attributions ranged from α = 0.58–0.74 and for other-attributions ranged from α = 0.87–0.90. Traditional methods for examining the NMSPI (i.e., at the scale and subscale levels) tend to report reliability levels of α ≥ 0.82 (Harms and Biocca, 2004), although to the best of our knowledge, no efforts to date have reported the reliability results with this self- and other-distinction. As such, it difficult to say whether the lower reliability for self-attributions tends to be the general case with the NMSPI.

#### Analysis of social presence

To analyze the effects of the independent variables on measures of social presence, we then conducted a 3 (gaze) × 2 (proxemic behavior) × 2 (measurement time) × 2 (focus of the attribution) mixed-model ANOVA, with gaze as the between-subjects variable (congruent, human-oriented, or variable), and proxemic behavior (assertive or passive), time of NMSPI measure (after trial 1 or after trial 6 for a given proxemic behavior) and focus of the attribution in the NMSPI (self- and other-), as the within-subjects variables.

Results indicated a significant main effect for proxemic behavior [*F*_(1,69)_ = 8.08, *p* = 0.006, $\eta_{p}^{2}$ = 0.11], such that the mean score for the Passive behavior (*M* = 3.69) was greater than the mean for the assertive behavior (*M* = 3.53). There was also a significant main effect for self- and other-dimensions of the NMSPI**[*F*_(1,69)_ = 57.66, *p* < 0.001, $\eta_{p}^{2}$ = 0.46], such that mean scores for the self-attributions were greater (*M* = 3.81) than for other-attributions (*M* = 3.42). Finally, the analyses showed a significant main effect for time of measure [*F*_(1,69)_ = 15.10, *p* < 0.001, $\eta_{p}^{2}$ = 0.18], such that mean scores for the first time (*M* = 3.53) were lower than the mean scores for the second time (*M* = 3.70).

**FIGURE 3:**
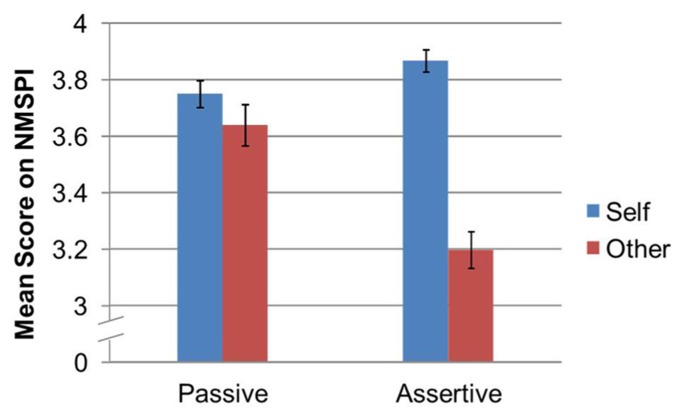
**Interaction between proxemic behavior condition and mean scores for self- and other-attributions as measured by the NMSPI**.

The main effects were further explained by a significant two-way interaction effect between proxemic behavior and the NMSPI dimensions of self- and other- [*F*_(1,69)_ = 42.87, *p* < 0.001, $\eta_{p}^{2}$ = 0.38]. Follow-up pairwise comparisons using Bonferroni corrections were conducted to better understand the interaction between proxemic behavior and the self- and other-dimensions (refer to **Figure 3**). Specifically, there was a significant difference among self-attributions between the assertive proxemic behavior condition (*M* = 3.87, SE = 0.04) and the passive behavior condition (*M* = 3.75, SE = 0.05, *p* = 0.009). There were also significantly lower scores among the other-attributions in the assertive proxemic condition (*M* = 3.20, SE = 0.06) when compared to the passive condition (*M* = 3.64, SE = 0.07, *p* < 0.001).

In order to help address our research question regarding the NMSPI, we also report on the pairwise comparisons between the self- and other-attributions. Specifically, the difference between the self- and other-attributions for the assertive proxemic behavior was significant, *p* < 0.001, but there was no significant difference between the self-attributions and other-attributions for the passive proxemic behavior, *p* = 0.119.

**FIGURE 4:**
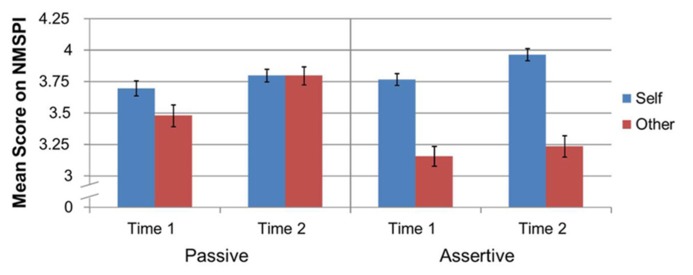
**Three-way interaction between proxemic behavior condition, time of measure, and mean scores for self- and other-attributions as measured by the NMSPI**.

Finally, a significant three-way interaction was found between proxemic behavior, NMSPI dimensions of self- and other-, and time of measure [*F*_(1,69)_ = 8.25, *p* = 0.005, $\eta_{p}^{2}$ = 0.11]. We conducted follow-up pairwise comparisons with Bonferroni corrections for the three-way interaction between proxemic behavior conditions, the self and other dimensions, and time of measure (refer to **Figure 4** for visual comparison).

Examining the differences between the proxemic behavior conditions revealed that there were no significant differences for the self-attributions at the first measure time between the passive (*M* = 3.70, SE = 0.06) and assertive (*M* = 3.77, SE = 0.05) proxemic behaviors, *p* = 0.301. Significant differences were found, however, for all other comparisons. At the second time of measure, self-attribution ratings were higher in the assertive condition (*M* = 3.97, SE = 0.05) compared to the passive condition (*M* = 3.80, SE = 0.05), *p* = 0.002. Other-attributions were higher during the first time of measure for the passive condition (*M* = 3.48, SE = 0.08) when compared to the assertive condition (*M* = 3.16, SE = 0.08), *p* = 0.004. Comparisons of other-attributions at the second time of measure revealed significantly lower ratings for the assertive condition (*M* = 3.24, SE = 0.09) than for the passive condition (*M* = 3.80, SE = 0.07), *p* < 0.001. These results show that when the robot was behaving according to the assertive proxemic behavior, self-attributions of the robot were higher than when the robot behaved passively, but only at the second measurement time. Conversely, other-attributions to the robot were higher in the passive condition than the assertive condition at both measurement times.

These analyses revealed differential changes in perceived attributions over repeated interactions based on the proxemic behavior expressed by the robot. For the passive proxemic behavior, there was no significant difference for the self-attributions between the first measure (*M* = 3.70, SE = 0.06) and the second measure (*M* = 3.80, SE = 0.05), *p* = 0.082, though significant differences were found for the other-attributions during the passive behavior between the first measure (*M* = 3.48, SE = 0.09) and the second measure (*M* = 3.80, SE = 0.07), *p* < 0.001. For the assertive proxemic behavior, there was a significant increase in self-attributions from the first measure (*M* = 3.77, SE = 0.05) to the second measure (*M* = 3.97, SE = 0.05), *p* < 0.001; however, there was no significant difference for other-attributions between the first measure (*M* = 3.16, SE = 0.08) to the second measure (*M* = 3.24, SE = 0.09), *p* = 0.457. These results show that in the passive condition, self-attributions of the robot remained stable across repeated interactions, but there were increases in the other-attributions. Conversely, when the robot behaved assertively there was an increase across the interactions for the self-attributions, but not for the other-attributions to the robot.

This interaction can also help address our research question about the utility of the NMSPI as a measurement instrument to assess mental state attribution. For the passive proxemic behavior, participant ratings for other-attributions at the time of first measure were significantly lower (*M* = 3.48, SE = 0.09) than the self-attribution ratings (*M* = 3.70, SE = 0.06, *p* = 0.007). However, at the time of the second measure in the passive condition, ratings for self-attributions and other-attributions were not significantly different (*M* = 3.80, SE = 0.07 and *M* = 3.80, SE = 0.05 for other and self respectively, *p* = 0.966). During the assertive proxemic behavior condition, participant ratings were significantly lower for the other-attributions (*M* = 3.16, SE = 0.08) than for the self-attributions (*M* = 3.77, SE = 0.05, *p* < 0.001). This remained true for the second measure in the assertive condition (*M* = 3.24, SE = 0.09 and *M* = 3.97, SE = 0.05 for other-attributions and self-attributions respectively, *p* < 0.001). These results show that when the robot was behaving according to the passive proxemic behavior condition, differences in self-attributions of the robot and other-attributions to the robot slowly disappeared over repeated interactions while the difference remained stable over repeated interactions when the robot behaved according to the assertive behavioral programming.

The predicted effect of gaze on perceived social presence was not significant, [*F*_(2,69)_ = 0.17, *p* = 0.846, $\eta_{p}^{2}$ = 0.01] and the predicted interaction between gaze and proxemic behavior was also not significant [*F*_(2,69)_ = 2.66, *p* = 0.078, $\eta_{p}^{2}$ = 0.07]. Thus, there was no support for H1_a_ and H3_a_. All other interactions were also non-significant, all *F *≤ 2.01, all *p* ≥ 0.141, $\eta_{p}^{2}$ = 0.054.

### EMOTIONAL STATE ATTRIBUTIONS TO THE ROBOT

In order to examine our hypotheses concerning emotional states attributed to the robot (H1_b_, H2_b_, H3_b_, and H4_b_), we next report a series of analyses to understand the impact of proxemic behavior, gaze, and time on the CMS. The results of emotional state attributions to the robot were first examined along the two dimensions of valence and arousal, as captured by the CMS. Given that participants responded to the CMS by clicking on an image presented on a computer screen, responses were saved as raster coordinates as a function of the pixel location on the image. We first converted these raster coordinates to Cartesian coordinates in order to analyze the dimensions of valence and arousal, where valence was represented by the *x*-axis values and arousal was represented by the *y*-axis values. For this conversion, the center point of the CMS image was converted to (0, 0) and the perimeter converted to encompass a space between -1 and +1 along both the *x*- and *y*-axes.

We then conducted a 3 (gaze) × 2 (proxemic behavior) × 2 (measurement time) × 2 (CMS dimension) mixed-model ANOVA, with gaze as the between-subjects variable (congruent, human-oriented, or variable), and proxemic behavior (assertive or passive), time of CMS measure (after trial 1 or after trial 6 for a given proxemic behavior) and CMS dimensions (valence and arousal), as the within-subjects variables. Results showed a significant main effect for the two CMS dimensions [*F*_(1,69)_ = 16.02, *p* < 0.001, $\eta_{p}^{2}$ = 0.19], such that the dimension of *arousal* (*M* = 0.21) was rated as higher than the dimension of *valence* (*M* = -0.01, *p* < 0.001), and a significant main effect for time of measure [*F*_(1,69)_ = 4.11, *p* = 0.047, $\eta_{p}^{2}$ = 0.06], such that the *second* measure time (*M* = 0.13) had higher ratings than the *first* measure time (*M* = 0.07).

**FIGURE 5:**
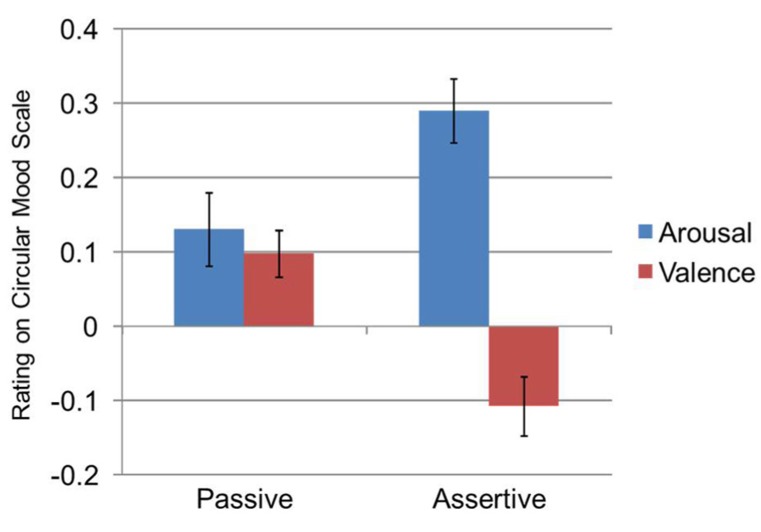
**Interaction between proxemic behavior condition and the dimensions of mood as measured by the CMS**.

The main effects were further explained by an interaction effect between proxemic behavior and the CMS dimensions, [*F*_(1,69)_ = 21.20, *p* < 0.001, $\eta_{p}^{2}$ = 0.24]. Although the predicted *main* *effect* of proxemic behavior was non-significant, [*F*_(1,69)_ = 0.66, *p* = 0.418, $\eta_{p}^{2}$ = 0.01], follow-up pairwise comparisons within the interaction, using Bonferroni correction, examined the interaction between the two proxemic behavior conditions and the two CMS dimensions. Results showed that *valence* was significantly greater during the passive proxemic behavior condition (*M* = 0.10, SE = 0.03) when compared to the assertive condition (*M* = -0.11, SE = 0.04, *p* < 0.001). Further, *arousal* was significantly greater during the assertive proxemic behavior condition (*M* = 0.29, SE = 0.04) when compared to the passive condition (*M* = 0.13, SE = 0.05, *p* = 0.005). From these results and examination of **Figure 5**, participants seemed to rate the emotion of the robot as reflecting more positive valence and a lower level of arousal when in the passive condition; whereas, in the assertive condition, participants seemed to rate the emotion of the robot with higher arousal and a negative valence. These findings lend partial support to H2_b._

The predicted main effect of gaze was not significant [*F*_(2,69)_ = 1.87, *p* = 0.158, $\eta_{p}^{2}$ = 0.05]. The predicted interaction between proxemic behavior and gaze was also not significant [*F*_(2,69)_ = 0.86, *p* = 0.426, $\eta_{p}^{2}$ = 0.02]. All other results were non-significant and irrelevant to our hypotheses, all *F *≤ 2.87, all *p* ≥ 0.08, $\eta_{p}^{2}$ ≤ 0.01.

A subsequent mixed-model ANOVA examined the results of CMS responses on a third dimension, intensity, which reflects the strength of the rating regardless of its valence or arousal. To do so, we converted participant responses from the Cartesian coordinates described above to Polar coordinates and the radius value or the distance from the origin represented intensity. For this 3 (gaze) × [2 (proxemic behavior) × 2 (measurement time)] mixed-model ANOVA, gaze (congruent, human-oriented, or variable) was the between subjects variable, and proxemic behavior (assertive or passive) and time of measure (after trial 1 or trial 6 for a given proxemic behavior) were used as within-subjects variables. Results showed a main effect for proxemic behavior [*F*_(1,69)_ = 4.23, *p* = 0.044, $\eta_{p}^{2}$ = 0.06], such that the ratings in the assertive condition (*M* = 0.65) were more intense than the passive condition (*M* = 0.60). This finding also lends partial support to H2_b._

**FIGURE 6:**
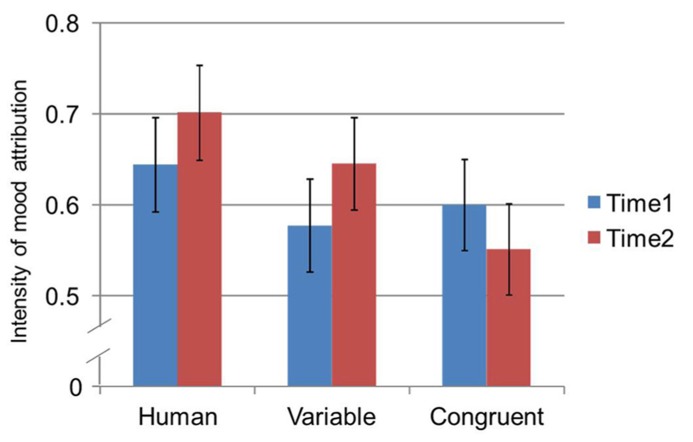
**Two-way interaction between measurement time and gaze on intensity of emotional attributions measured by the CMS**.

Further, a significant interaction was found between time and gaze [*F*_(2,69)_ = 3.70, *p* = 0.030, $\eta_{p}^{2}$ = 0.10]. Despite the significant two-way interaction between measurement time and gaze on the intensity of emotional attributions, follow-up pairwise comparisons with Bonferroni corrections did not reveal any significant difference within the three levels of gaze at either the first and second measurement times (all *p* > 0.05). By contrast, when comparing each of the levels of gaze between the first and second measurement times (see **Figure 6**), results of pairwise comparisons with Bonferroni corrections indicated that, for *variable* gaze only, the level of intensity was significantly greater during the second measurement time (*M* = 0.65, SE = 0.05) when compared to the first measurement time (*M* = 0.58, SE = 0.05, *p* = 0.05). These results indicate that while there were no differences across the gaze types and measurement times, there was a difference within the variable gaze condition that showed an increase in intensity across repeated interactions.

**FIGURE 7:**
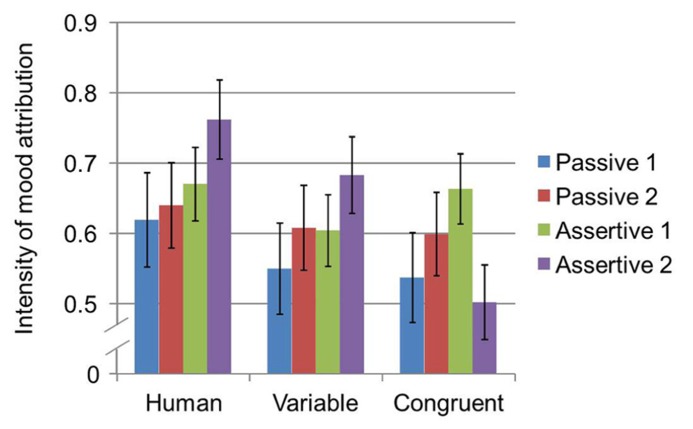
**Three-way interaction between proxemic behavior, measurement time, and gaze on intensity of emotional attributions measured by the CMS**.

Lastly, a three-way interaction was found between proxemic behavior, time, and gaze [*F*_(2,69)_ = 6.90, *p* = 0.002, $\eta_{p}^{2}$ = 0.17] on intensity (see **Figure 7**). Tests of simple effects were computed in order to conduct pairwise comparison between each of the three variables at all levels. Results showed that, for assertive proxemic behavior during the second time of measurement, intensity for human gaze (*M* = 0.76, SE = 0.06) was significantly greater than congruent gaze (*M* = 0.50, SE = 0.05, *p* = 0.004). Additionally, for human gaze during the second measurement time, intensity was significantly greater during the assertive proxemic behavior condition (*M* = 0.76, SE = 0.06) when compared to the passive condition (*M* = 0.64, SE = 0.06, *p* = 0.023). By contrast, for congruent gaze during the first measurement time, intensity was significantly greater during the assertive proxemic behavior condition (*M* = 0.66, SE = 0.05) when compared to the passive condition (*M* = 0.54, SE = 0.06, *p* = 0.032). Lastly, for congruent gaze during assertive proxemic behavior, intensity significantly decreased from the first time it was measured (*M* = 0.66, SE = 0.05) when compared to the second time it was measured (*M* = 0.50, SE = 0.05, *p* = 0.001).

From these results and examination of **Figure 7**, this interaction seems to be primarily driven by differences in the patterns between the first and second measurement during assertive proxemic behavior for the congruent gaze. Specifically, the figure shows that, across the three gaze variables, there were increases in intensity attributions between the first and second measurements in both proxemic behavior and for two of the three gaze conditions, but not in the congruent gaze condition. Here, rather than an increase in intensity of emotional attributions between the first and second assertive measurement, a decrease in intensity occurred. These interaction effects with gaze, thus, provided partial support for H3_b_ and H4_b_.

## DISCUSSION

The purpose of this research was to investigate how social cues (gaze and proxemic behavior), instantiated in a mobile robot platform, affected perceived social presence of the robot and how humans would interpret those cues as social signals. Additionally, this study examined changes in perceptions of a robot over multiple interactions. Overall, we found that proxemic behavior affected perceptions of the robot as a socially present agent and led to different attributions of emotions. Specifically, participants considered a robot that acted passively as a more socially present agent than a robot that acted assertively. Participants also rated the valence of emotions attributed to the robot in the passive condition as more positive than those of the assertive robot; and they rated the arousal level attributed to the robot in the assertive condition higher than in the passive condition. Perceptions of social presence and emotional states also tended to increase over repeated interactions, regardless of the robot’s gaze or proxemic behavior programming.

In support of hypothesis H2_a_, findings from this experiment regarding social presence suggest that, when the robot behaved in accordance with the passive proxemic behavioral programming, the robot was perceived as a more socially present agent. This may be because the robot appeared to consider implicit social rules of politeness during the shared navigation situation (see Tsui et al., 2010). For example, giving the “right of way” when a path-crossing event occurs, as was the case in the passive proxemic behavior condition, may have led participants to perceive the robot as attending to them and their behavior. In HHIs, attending to and responding in a way that takes into consideration the interests of another has been termed “social mindfulness,” and appears to facilitate positive perceptions of an entity (Van Doesum et al., 2013).

The results of the experiment did not support H1_a_ or H3_a_, indicating that the gaze of the robot did not seem to be as important a social cue for this specific scenario. Instead, gaze only appeared to affect intensity of perceived emotional states when interacting with the cue of proxemic behavior in our path-crossing scenario. This implies that the manner in which the robot moved through space mattered more than where the robot was perceived to be looking during this particular shared navigation situation. These results provide further evidence for the notion that the design of robots intended for use in environments where social situations are likely should account for findings that certain social cues better afford the perception of a robot as a social agent. In doing so, it is likely that this can increase the degree to which people can appropriately interpret social signals; that is, attribute mental states to a robot and in turn, understand its intentions.

In support of H4_a_, the results also showed that the robot was perceived as more socially present over repeated interactions. As participants became more familiar with the robot behaving in a certain way (i.e., passive or assertive), they increasingly saw it as socially present and thus capable of having mental and emotional states. This increase in perceived social presence is, however, dependent on the social cues expressed and the context of the situation. For instance, in this study, the increase in perceived social presence for the passive proxemic behavior condition was greater than the increases for the assertive proxemic behavior condition. But the robot also displayed a change in behavior mid-way through the experiment that could also have contributed to this pattern of increase in perceived social presence. One implication of this is that, if humans are going to be required to interact with a robot over a period of time, the ways in which certain social cues are expressed will differentially affect the rate of increase in perceiving the robot as a social agent. Notably, the repeated interactions in this experiment still occurred over a relatively short period and, as such, claims cannot be made regarding whether these findings would hold true for repeated interactions that span days, weeks, or months as will be the case in real-world implementations of robotic systems.

### IMPLICATIONS

The results from this experiment provide advances in both theoretical and practical understanding of social signals research in the context of HRI as well as robotic design applications that can inform the intuitive exchange of social cues and signals essential to such interactions. This is particularly important given that many robots that are being deployed will be non-humanoid. Our research supports the notion that even non-humanoid robots can convey social cues and that they are interpreted as meaningful signals.

Our study also provides insights into understanding how to measure intentions and attributions. We suggest that social presence is an important component of gaining access to intentional and other mental states, particularly in the case of human interactions with artificial intelligences or technologically mediated others (Biocca and Harms, 2002). Additionally, the present study examined perceptions of emotional states of a robot as this can also be a key social signal providing information about the intentions of an agent.

The study’s findings generally suggest that different social cues elicit different emotional attributions, some of which may increase over repeated interactions. When programmed to behave more assertively, the robot was perceived as having emotional states that were indicative of higher states of arousal. In addition, the intensity of emotional state attributions were also greater when the robot behaved assertively, particularly when comparing the gaze behavior that was oriented to a person’s head with the gaze behavior that focused only on the direction of navigation. Conversely, when programmed to behave in a way that was more passive, more positive valence emotional attributions were made compared to when the robot acted assertive. Further, across repeated interactions, the social cues of robot gaze and proxemic behavior influenced whether the intensity of emotional attributions increased, as in the passive and assertive proxemic conditions with human or variable gaze, or decrease, as in the assertive proxemic condition with congruent gaze.

Taken together, these results support H2_b_, H3_b_, and H4_b_ and suggest that roboticists can leverage different social cues to convey certain emotional states that provide information about the intentions of a robot, regardless of whether that robot is capable of having emotional states or not. For example, if a robot should be regarded as having more positive emotional states, it should be programmed to appear as a socially mindful agent (see Tsui et al., 2010; Van Doesum et al., 2013). If the robot should be regarded as more active or more aroused, possibly conveying a sense of urgency, it should execute behaviors that appear more assertive and without as much, if any, regard for those implicit social rules.

We also examined how participants responded regarding their own perceptions of the robot (self-attributions) and how they believed the robot perceived and thought about the participant (other-attributions), as the latter requires engaging in theory of mind. Our results showed differential changes in perceived social presence and attributions of emotional states of mind depending on proxemic condition. We also found that differences in self-attributions of the robot and other-attributions to the robot slowly disappeared over repeated interactions when the robot was behaving according to the passive proxemic behavior condition. But, in the assertive condition, the differences remained stable over repeated interactions.

The current results provide support for the notion that endowing robots with the capability of expressing social cues, such as gaze behaviors and proxemic behaviors, results in them being explicitly considered as social agents, in line with prior research (e.g., Saulnier et al., 2011; Kahn et al., 2012a,b). One issue with this interpretation is that the present study did not include either a HHI condition or a human–computer interaction condition for comparison. It may be that socially capable non-humanoid robots are considered more socially present than a computer but less than a real human, or that such a robot is considered as socially present as a computer or a human. Future research in HRI is needed to examine where varying types of robots may lie on this continuum of potential social actors, in terms of social presence.

### LIMITATIONS

There are several limitations present in the design of this study. The context of the experiment, a shared navigation scenario within a relatively narrow hallway involving path crossing, limits the generalizability of our results. Subsequent studies would need to conduct research across other shared navigation situations to further tease apart the relationships between a robot’s expression of social cues and how these are interpreted as social signals. Second, the sample population in the present study consisted entirely of college undergraduates. As social robots are likely to be deployed across many contexts, it is crucial to understand how other populations perceive the robot. It is also worth noting that the present study only examined two cues, gaze, and proxemic behavior. While there were several findings relating to how proxemic behavior affects perception of the robot, the gaze cue was not significant. This finding could be due to the relatively weak manipulation of gaze in the present study. That is, the robot had a head without a face, and the entire head-unit moved as a function of the gaze programming. Many other studies that have found gaze to be an important non-verbal social cue examined it in robots with more robust facial features, such as eyes (e.g., Mutlu et al., 2009; Takayama and Pantofaru, 2009; Mumm and Mutlu, 2011; Saulnier et al., 2011).

One additional point needs to be made regarding our instructions to participants to respond using the CMS to attribute an emotional state to the robot. This could be construed as biasing the participants to believe the robot was truly capable of having emotional states. However, the present experiment was not intended to investigate whether or not people believe robots have emotions, but, instead, to examine what emotional states they felt the robot was exhibiting. Participants were informed during the training phase that the center point of the CMS was indicative of a neutral emotion, and, in fact, a number of our participants did select the central regions throughout the experiment. The cases where participants did attribute an emotional state to the robot as a function of the social cues displayed and repeated interactions were what were of primary interest to us.

## CONCLUSION

Despite the aforementioned limitations, it is important to iterate the specific contributions of the present study as these include an integrated set of factors. First, the experiment was conducted in a realistic setting in which participants had actual interactions with a robot programmed to convey multiple social cues. This is in contrast to the use of simulated or “man behind the curtain” type experiments that are prevalent in HRI. Next, we were able to examine changes over time as the participants interacted with the robot across multiple trials and responded to the measures multiple times. Additionally, many HRI experiments rely on observational data (e.g., the distance participants maintained from the robot) or subjective measures of participants’ attitudes about a given robot or robots in general. We used measures that assessed varying mental states that participants could attribute to a robot even though it, in fact, possessed no mental states. This leads to another novel contribution of the research in terms of the way in which the NMSPI was analyzed. We suggest that parsing the scale along the self- and other-dimensions is a unique contribution that should be pursued in future HRI studies. Lastly, the use of a multi-dimensional single-item measure for attributing emotional states (CMS) to a robot is a unique contribution and one that has broad applicability for not only research in HRI, but also HHI as well.

In closing, the expression of social cues by a robot determines how it is perceived as a social agent capable of having mental and emotional states. This is important to consider as robots transition not just from sterile, industrial settings to dynamic, social contexts, but also as robots transition from tools to potential team members (Fiore et al., 2008; Phillips et al., 2011; Wiltshire et al., 2013a). Roboticists can leverage findings such as the ones presented here, and those that build upon our efforts, to design robots that project cues enabling humans to make accurate mental state attributions and, potentially, predict robot actions as they interact in service of collaboratively meeting goals and completing tasks.

## Conflict of Interest Statement

The authors declare that the research was conducted in the absence of any commercial or financial relationships that could be construed as a potential conflict of interest.

## ACKNOWLEDGMENTS

This work was partially supported by the Army Research Laboratory and was accomplished under Cooperative Agreement Number W911NF-10-2-0016. The views and conclusions contained in this document are those of the authors and should not be interpreted as representing the official policies, either expressed or implied, of the Army Research Laboratory, the U.S. Government or the University of Central Florida. The U.S. Government is authorized to reproduce and distribute reprints for Government purposes notwithstanding any copyright notation herein. We thank Paul Wiegand for assistance in analyzing the data from the circular mood scale measures.
